# Characterization of the antibacterial activity of Bald’s eyesalve against drug resistant *Staphylococcus aureus* and *Pseudomonas aeruginosa*

**DOI:** 10.1371/journal.pone.0208108

**Published:** 2018-11-28

**Authors:** Amanda L. Fuchs, Alan J. Weaver, Brian P. Tripet, Mary Cloud B. Ammons, Martin Teintze, Valérie Copié

**Affiliations:** Department of Chemistry and Biochemistry, Montana State University, Bozeman, Montana, United States of America; Universitatsklinikum Munster, GERMANY

## Abstract

Bald’s eyesalve is an Anglo-Saxon medicinal remedy that has been used through ancient times to treat eye sty infections and may represent a source of ancientbiotics. This study assessed the efficacy of Bald’s eyesalve against several strains of *Staphylococcus aureus* and *Pseudomonas aeruginosa*, including a multi-drug resistant phenotype, and identified the principal compound conveying antibacterial activity. Bald’s eyesalve formulations were produced by combining garlic, onion or leek, wine, bovine bile, and brass, with specific ingredient omissions in several formulations, followed by incubation at 4 °C for 9 days. Bald’s eyesalve formulation ES-GBBr exhibited the greatest antibacterial activity against *S*. *aureus* and *P*. *aeruginosa*. Fractionation of ES-GBBr using molecular size exclusion and organic solvent partitioning isolated its antibacterial activity to the small molecule nonpolar fraction, and 1D ^1^H NMR revealed the identity of the antibacterial agent to be allicin. Depletion of allicin from this fraction by addition of exogenous cysteine established that all observable growth inhibition originated from allicin. Quantification of allicin demonstrated that its concentration was significantly greater in ES-GBBr compared to the ES-O formulation; however, this was not due to greater yield. The antibacterial activity of allicin against *S*. *aureus* was antagonized by other ingredients within Bald’s eyesalve, whereas they were additive or synergistic against *P*. *aeruginosa*. These results suggest that neither leek nor onion is necessary for the antibacterial efficacy of Bald’s eyesalve against *S*. *aureus* or *P*. *aeruginosa*, and while allicin was identified as the principal antibacterial agent present, its activity is influenced differentially in the presence of additional Bald’s eyesalve ingredients when used against *S*. *aureus* compared to *P*. *aeruginosa*. Ancientbiotics may provide a source of promising antibacterials; however, identifying the source of activity and assessing distinct formulations for cooperative effects are essential to using ancient remedies, such as Bald’s eyesalve, effectively against drug resistant pathogens.

## Introduction

Ancient Anglo-Saxon medicinal remedies have been receiving renewed scrutiny concerning their potential as rich sources of antibacterial compounds that could help combat modern drug-resistant bacterial infections [1]. As the discovery and development of novel antibiotics declines, the emergence of antibiotic resistant pathogens is an alarming threat to human health, with an estimated 700,000 deaths resulting from drug-resistant infections per year. Predictions indicate that such infections will cause 10 million deaths annually by the year 2050 if new antimicrobial compounds are not developed soon [2]. While ancient medical formulations, such as Bald’s eyesalve, are being revisited for their antibacterial potency, little is known regarding the specific agents responsible for their efficacy, the cooperative effects within these complex mixtures, or whether such natural remedies could be utilized to effectively address the threat of modern antibiotic resistant microbes.

The development of bacterial resistance to modern antibiotics is startling and has thus prompted the investigation of ancient medieval remedies for promising antibiotic compounds. Bald’s eyesalve originates from a 10^th^ century English medicinal text, *Bald’s Leechbook*, and has been used to treat bacterial infections of the eyelash follicle, commonly known as a sty. The formulation consists of wine, garlic, an additional *Allium* species, such as onion or leek, and bovine bile; these ingredients are combined within a brass or bronze vessel and left to incubate for 9 days prior to use [3]. Common pathogens found in modern bacterial eye infections include *Staphylococcus aureus* and *Pseudomonas aeruginosa*, including methicillin-resistant *S*. *aureus* (MRSA) and drug resistant *P*. *aeruginosa* strains. In addition, these pathogens are responsible for a wide variety of severe, chronic infections, including sepsis, pneumonia, and chronic wound infections [4, 5].

In a 2015 study, Bald’s eyesalve was found to exhibit bactericidal activity against MRSA biofilms in an *in vitro* synthetic wound model; however, its mechanism of action and the antimicrobial agent(s) responsible for its efficacy were never elucidated [6]. In the present study, we sought to identify the principal antibacterial compound within Bald’s eyesalve using various formulations, a multi-step fractionation process, 1D ^1^H NMR analysis, and minimum inhibitory concentration (MIC) assays. Furthermore, we aimed to establish the efficacy of Bald’s eyesalve against both Gram-positive and Gram-negative bacteria, such as *S*. *aureus* and *P*. *aeruginosa*, including the MRSA strain LAC and clinical wound isolate *P*. *aeruginosa* PA215.

## Materials and methods

### Bald’s eyesalve materials

The garlic (*Allium sativum*), yellow onion (*Allium cepa* L.), and leek (*Allium ampeloprasum* L.) used in this study originated from Montana Stinking Rose Farm in Bozeman, Montana, Easterday Farms Produce Company in Pasco, Washington, and Associated Food Stores Inc. in Salt Lake City, Utah, respectively. Plant materials and white wine (2016 Pinot Grigio, La Fiera Veneto; UPC 0-89832-90007-8) were purchased from Town & Country Foods in Bozeman, Montana. Following purchase, produce and wine were stored at 4 °C prior to Bald’s eyesalve preparation.

Bovine bile salts were purchased from Sigma Aldrich (product no. B3883; lot no. SLBH1798V), and 0.51-mm brass sheet (24 gauge; alloy 260) was purchased from Ace Hardware in Bozeman, Montana (product no. 5024864).

### Preparation of crude Bald’s eyesalve

Bald’s eyesalve formulations (Table 1) were prepared as previously described [6] unless noted otherwise. In brief, garlic bulbs and yellow onion were peeled and coarsely chopped prior to being minced in a food processor (Mainstays Food Chopper, model no. FPMEMC3002). Leeks were chopped in half, and the leeks greens were coarsely chopped and the minced using a food processor as described above, while the white root end of the leek was discarded.

**Table 1:** Composition of Bald’s eyesalve (ES) formulations.

	ES-O	ES-L	ES-GBBr	ES-GB	ES-GBr	ES-G
Garlic	+	+	+	+	+	+
Wine	+	+	+	+	+	+
Onion	+	-	-	-	-	-
Leek	-	+	-	-	-	-
Bovine bile^a^	+	+	+	+	-	-
Brass^b^	+	+	+	-	+	-

Bald’s eyesalve was prepared by combining equal volumes (25 mL) of garlic, wine, onion or leek, and bovine bile followed by addition of brass prior to incubation at 4 °C for 9 days. + designates ingredients that were included, while - denotes those which were excluded.

^a^87 mg/mL dissolved in sterile water

^b^Nine 15-mm squares of 24 gauge (0.51-mm) brass sheet metal (alloy 260)

Immediately following plant material preparation, equivalent volumes (25 mL) of minced garlic bulb (~14.2 g), minced yellow onion (~22.2 g) or minced leek leaves (~8.8 g), white wine, and bovine bile salts (87 mg/mL dissolved in sterile water) were combined in sterile 250 mL glass bottles. Nine 15-mm squares of 0.51-mm brass sheet were added to each bottle prior to being wrapped in foil and placed at 4 °C for 9 days. One or more ingredients were omitted from several formulations as described in Table 1. After incubation, formulations were centrifuged at 5000 rpm for 10 min. Supernatants were transferred to sterile 50 mL tubes and stored at 4 °C, while insoluble materials were discarded.

### Bacterial strains

Methicillin-susceptible *S*. *aureus* (MSSA) strain ATCC 6538 [7] was purchased commercially. Community-associated methicillin-resistant *S*. *aureus* (CA-MRSA) strain LAC, pulsed-field gel-electrophoresis type USA300 [8] was provided by Dr. Jovanka M. Voyich. *P*. *aeruginosa* wild-type strain PAO1 [9] and clinical wound isolate strain PA215 [10] were provided by Dr. Michael J. Franklin and Dr. Garth A. James, respectively.

### Determination of minimal inhibitory concentration

MIC values were determined for each of the bacterial strains using a microdilution method in 96-well microtiter plates with cation-adjusted Mueller-Hinton broth (MHB). Inoculum culture growth conditions consisted of batch cultures grown at 37 °C, 220 rpm agitation to an optical density reading at 600 nm of 0.45–0.5 for *S*. *aureus* (~ 2.5 × 10^8^ colony-forming units per mL (CFU/mL)) and 0.48 for *P*. *aeruginosa* (~ 3 × 10^8^ CFU/mL). Inoculum was diluted 1:200 (v/v) with 2X MHB and then 1:1 (v/v) into 96-well microtiter plates containing serial diluted eyesalve (40% to 0.3125% (v/v)) along with positive and negative control wells, containing no eyesalve with and without inoculum respectively, for a total of 200 μL per well. Microtiter plates were incubated at 37 °C for 20 hrs prior to evaluation for growth inhibition. This method was based upon recommendations from the Clinical and Laboratory Standards Institute [11]. An allicin standard (LKT Labs) was assayed using the same protocol, but with *S*. *aureus* stain LAC and *P*. *aeruginosa* strain PA215 only.

### Molecular size exclusion and solvent partitioning fractionation of Bald’s eyesalve

The ES-GBBr formulation, consisting of garlic, wine, bovine bile, and brass, was centrifuged at 21000 x g for 10 min to remove any residual debris prior to fractionation. An aliquot of resultant supernatant was removed and stored at 4 °C as crude eyesalve, while the remaining supernatant was fractionated as described below.

Small molecule (SM) fraction was prepared by means of filtering 40 ml of the initially prepared crude eyesalve through a pre-washed Amicon Ultra-15 (cellulose) centrifugal filter unit with a 3 kDa molecular weight cut-off by centrifugation at 4000 x g, 4 °C for 60 min. The proteinaceous (PRT) fraction was prepared by restoring concentrate volume to 40 ml with sterile water. Both SM and PRT fractions were stored at 4 °C.

Small molecule, polar (SM-P) and nonpolar (SM-NP) fractions were prepared by combining 6 ml of SM fraction with 3.6 ml of chloroform. This mixture was vortexed for 1 min prior to phase separation by centrifugation at 5000 rpm for 10 min. The polar phase was removed and stored at 4 °C. The nonpolar phase was dried under a stream of N_2_ gas and reconstituted in 6 ml of sterile water prior to being stored at 4 °C.

### NMR sample preparation

Bald’s eyesalve formulations/fractions prepared as described above were centrifuged at 21000 rpm for 10 min to remove insoluble debris. A 300 μL aliquot of resultant supernatant was added to 300 μL of 2X NMR stock buffer (50 mM NaH_2_PO_4_/Na_2_HPO_4_, 0.8 mM imidazole as a pH indicator, and 0.5 mM 4,4-dimethyl-4-silapentane-1-sulfonic acid (DSS) in 20% D_2_O, pH 7) and then transferred to a 5 mm Bruker NMR tube.

### NMR experiments

All NMR spectra were collected at 298 K (25 °C) on a Bruker 600 MHz (^1^H Larmor frequency) AVANCE III solution NMR spectrometer, equipped with an automatic sample loading system (SampleJet), a 5 mm triple resonance (^1^H, ^15^N, ^13^C) liquid-helium-cooled TCI NMR probe (Cryoprobe), and Topspin software (Bruker version 3.1).

1D ^1^H NMR experiments were performed using the Bruker “zgesgp” pulse sequence and recorded with 256 scans and a ^1^H spectral window of 9615.38 Hz. Free induction decays were collected with 32K data points and a dwell time interval of 52 μsec amounting to a data acquisition time of ~ 1.7 s, and a 1 s delay between acquisitions, which resulted in an overall ~ 2.7 sec relaxation recovery delay between scans. 1D ^1^H NMR spectra were phase-corrected using Topspin software (Bruker version 3.1), and baseline correction was applied following import of the NMR spectra into the Chenomx NMR Suite program (version 8.0; Chenomx, Inc., Alberta, Canada). Subsequently, a recorded 1D ^1^H NMR spectrum of an allicin standard was added to the Chenomx small-molecule library for 600 MHz (^1^H Larmor frequency) magnetic field strength NMR using the ‘Compound Builder’ module of the Chenomx NMR Suite program (version 8.0) and was used as a reference spectrum for this compound. Allicin and ethanol were identified and quantified using the ‘Profiler’ module of Chenomx for each Bald’s eyesalve formulation and/or fraction. The internal DSS (0.25 mM) standard was used for quantification of metabolite concentrations.

### Depletion of allicin

An aliquot of the SM-NP fraction of the ES-GBBr formulation was titrated with L-cysteine to a final concentration of 15 mM and incubated at 4 °C for 1 hr. Allicin standard (0.5 mM), diluted from stock using sterile water, was also incubated in the presence of 15 mM L-cysteine under the same conditions, and resultant samples were analyzed by 1D ^1^H NMR.

### Statistical analysis

Data analysis was performed on 4 independent replicates of each Bald’s eyesalve formulation. Statistical significance was assessed by one-way analysis of variance (ANOVA), followed by Tukey’s multiple comparisons post-test using GraphPad Prism program version 7.1 (GraphPad Software, La Jolla, CA).

## Results

### Antibacterial efficacy of Bald’s eyesalve formulations against *S*. *aureus* and *P*. *aeruginosa*

The antibacterial activity of four different Bald’s eyesalve formulations, prepared as described in Table 1, was assessed against two strains of *S*. *aureus* and *P*. *aeruginosa*, including LAC and PA215. This initial formulation selection included those denoted as ES-O, ES-L, ES-GBBr, and ES-GB (Table 1). All crude Bald’s eyesalve formulations predominantly demonstrated strong growth inhibition for *S*. *aureus* strains ATCC 6538 and LAC with MIC values ranging from 0.625 to 2.5% (v/v) (Table 2). ES-O, ES-L, and ES-GB largely exhibited weak activity towards *P*. *aeruginosa* strains PAO1 and PA215 with MIC values ranging from 2.5 to 10% (v/v); however, the ES-GBBr formulation displayed moderate growth suppression for both *P*. *aeruginosa* strains with an MIC value of 2.5% (v/v) (Table 2).

**Table 2:** Susceptibility of bacterial strains to crude Bald’s eyesalve formulations.

Bacterial strains	MIC^a^			
	ES-O	ES-L	ES-GBBr	ES-GB
*S*. *aureus*				
ATCC 6538	0.625	0.625	0.625	0.625
LAC	2.5	1.25	1.25	1.25
*P*. *aeruginosa*				
PAO1	5	5	2.5	2.5
PA215	10	5	2.5	5

MIC was determined using a broth microdilution method as described by the Clinical and Laboratory Standards Institute. Reported values represent the median of at least 3 biological replicates.

^a^MIC values indicate the concentration of Bald’s eyesalve formulation in % (v/v)

### Isolation of antibacterial activity present in ES-GBBr formulation by fractionation

The ES-GBBr formulation was fractionated using molecular size exclusion into a proteinaceous (PRT) and small molecule (SM) fraction, and the antibacterial activity of these fractions was evaluated against the aforementioned bacterial strains. The PRT fraction showed notably weak activity against all strains with MIC values ranging from 10 to greater than 40% (v/v); furthermore, this reflected an MIC increase of 16-fold for *S*. *aureus* strain ATCC 6538, greater than 16-fold for *S*. *aureus* strain LAC, and greater than 32-fold for *P*. *aeruginosa* strains PAO1 and PA215 when compared to crude ES-GBBr (Table 3). The SM fraction demonstrated strong growth inhibition activity for *S*. *aureus* strains ATCC 6538 and LAC with MIC values ranging from 0.625 to 1.25% (v/v), and moderate to weak activity towards *P*. *aeruginosa* strains PAO1 and PA215 with MIC values ranging from 2.5 to 5% (v/v) (Table 3). These results indicated that the SM fraction sustained full antibacterial activity relative to crude ES-GBBr against all strains, except for *P*. *aeruginosa* strain PA215 which exhibited a 2-fold MIC increase (Table 3).

**Table 3:** Susceptibility of bacterial strains to ES-GBBr formulation fractions.

Bacterial strains	MIC^a^ (FC^b^)			
	PRT	SM	SM-P	SM-NP
*S*. *aureus*				
ATCC 6538	10 (16)	0.625 (1)	10 (16)	1.25 (2)
LAC	>40 (>32)	1.25 (1)	20 (16)	2.5 (2)
*P*. *aeruginosa*				
PAO1	>40 (>16)	2.5 (1)	40 (16)	5 (2)
PA215	>40 (>16)	5 (2)	>40 (>16)	10 (4)

MIC was determined using a broth microdilution method as described by the Clinical and Laboratory Standards Institute. Reported values represent the median of at least 3 biological replicates.

^a^MIC values indicate the concentration of ES-GBBr formulation fraction in % (v/v)

^b^FC values indicate the fold change in the MIC relative to that of crude ES-GBBr

Organic solvent partitioning was used to further fractionate the SM fraction of the ES-GBBr formulation into a small molecule polar and small molecule nonpolar fraction, and the antibacterial activity of these fractions was evaluated against all bacterial strains. The SM-P fraction showed weak activity against all strains with MIC values ranging from 10 to greater than 40% (v/v); correspondingly, this reflected an MIC increase of 16-fold for *S*. *aureus* strains ATCC 6538 and LAC, 16-fold for *P*. *aeruginosa* strain PAO1, and greater than 8-fold for *P*. *aeruginosa* strain PA215 when compared to the SM fraction (Table 3). The SM-NP fraction demonstrated strong to moderate growth inhibition activity for *S*. *aureus* strains ATCC 6538 and LAC with MIC values ranging from 1.25 to 2.5% (v/v), and weak activity towards *P*. *aeruginosa* strains PAO1 and PA215 with MIC values ranging from 5 to 10% (v/v) (Table 3). These results indicated that the SM-NP fraction sustained partial antibacterial activity, relative to the SM fraction, demonstrating a 2-fold MIC increase against all bacterial strains (Table 3).

### Identification and validation of the principal antibacterial agent in Bald’s eyesalve

**Fig 1:**
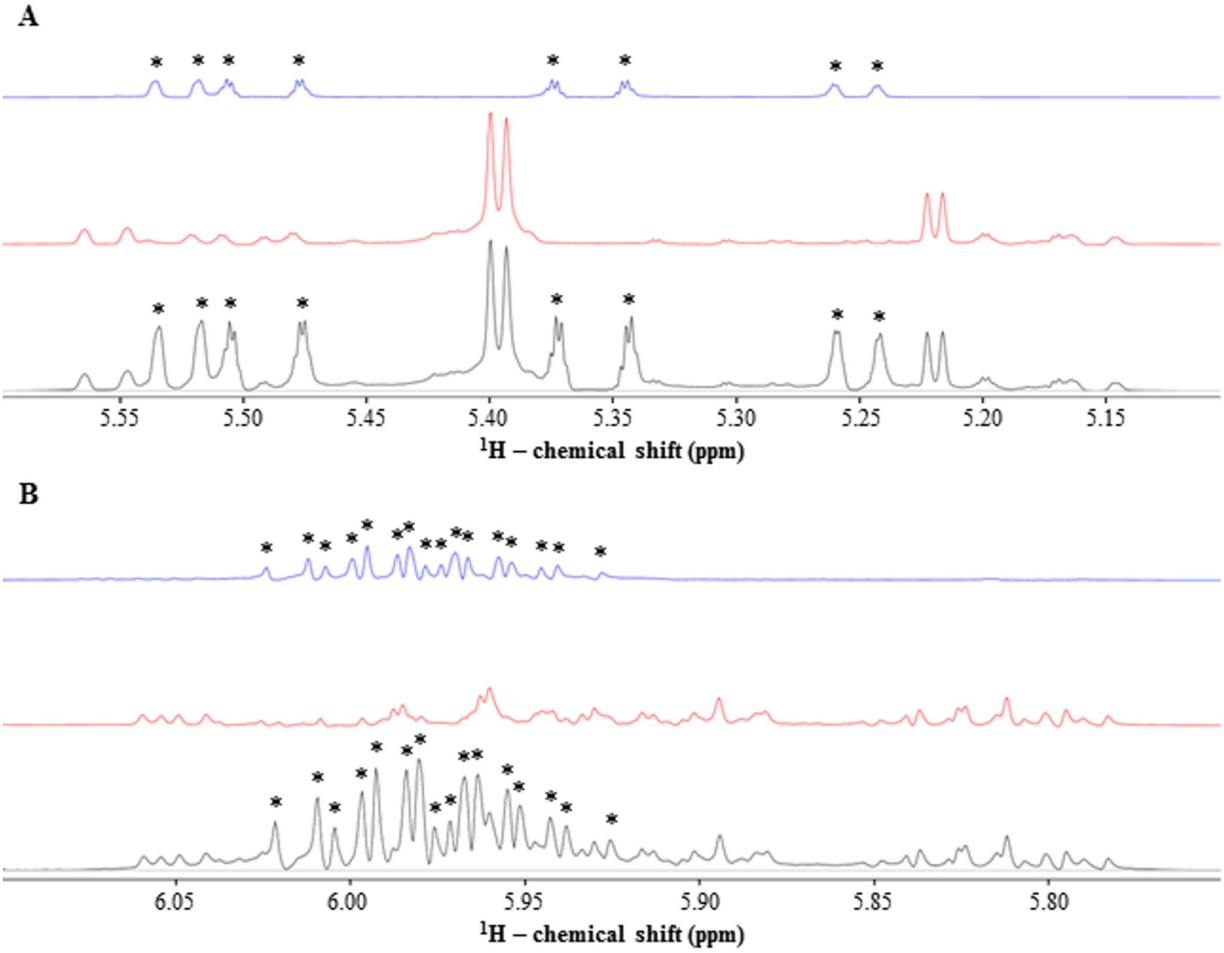
^1^H NMR analysis of ES-GBBr formulation fractions establishes presence of unknown compound(s). Stacked 1D ^1^H NMR spectra (600 MHz, 10% D_2_O) in chemical shift regions (A) 5.1–5.6 ppm and (B) 5.75–6.1 ppm for ES-GBBr SM (black), SM-P (red), and SM-NP (blue) fractions. Asterisks indicate NMR signals from molecule(s) of interest.

**Fig 2:**
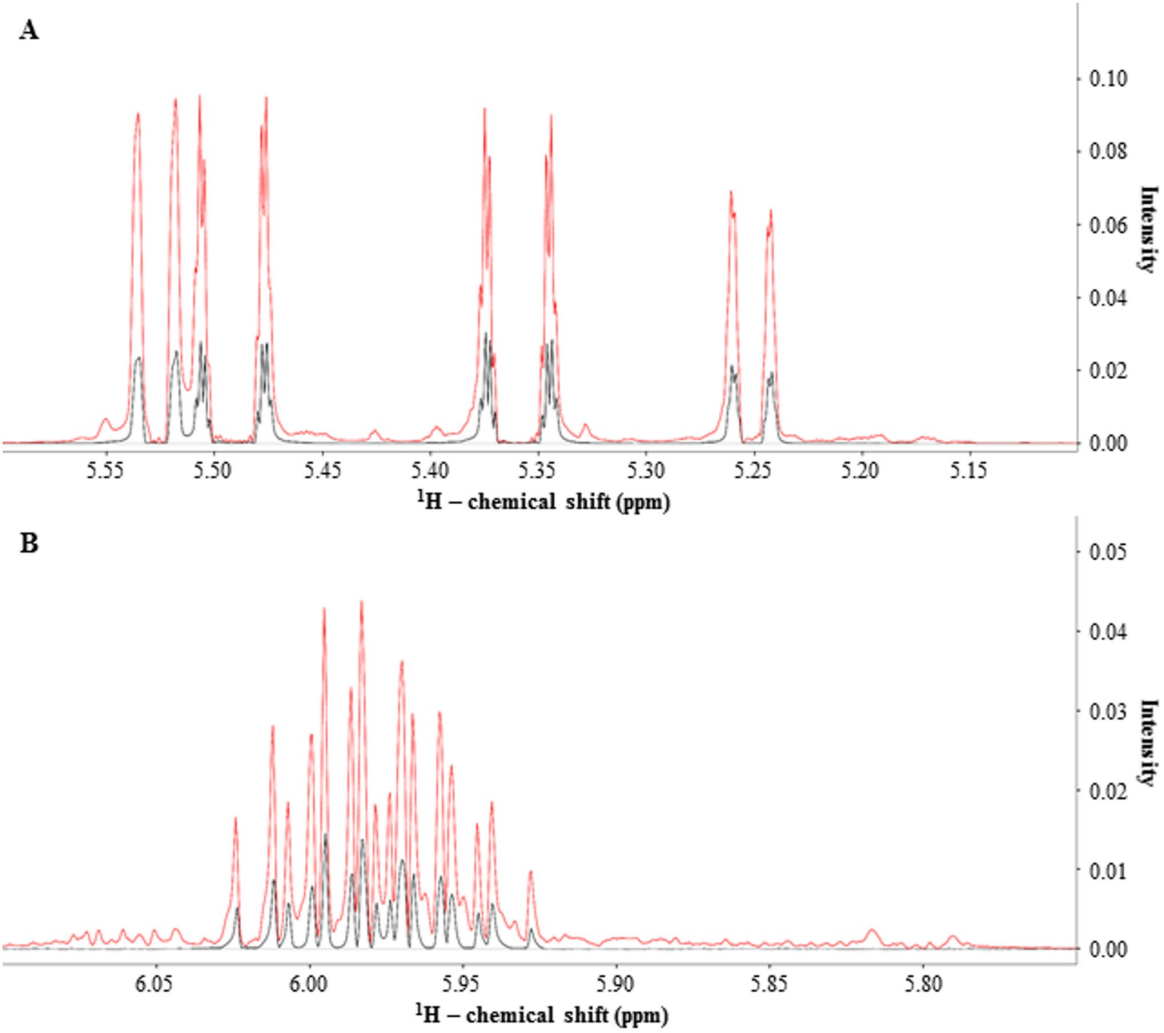
Identity of unknown determined to be an organosulfur compound originating from garlic known as allicin. An overlay of 1D ^1^H NMR spectra (600 MHz, 10% D_2_O) in chemical shift regions (A) 5.1–5.6 ppm and (B) 5.75–6.1 ppm for an allicin standard (black) and ES-GBBr formulation SM-NP fraction (red).

To identify the principal antibacterial agent in Bald’s eyesalve, 1D ^1^H NMR spectra of the SM, SM-P, and SM-NP fractions of the ES-GBBr formulation were recorded (S1 Fig). These 1D ^1^H NMR spectra revealed notable NMR signals within the 5.1 to 6.1 ppm chemical shift range that were present in the SM and SM-NP fractions while absent in the SM-P fraction (Fig 1). A 1D ^1^H NMR spectrum of an allicin standard was recorded, and this spectrum correlated with the NMR signals observed within the 5.1 to 6.1 ppm chemical shift range in the 1D ^1^H NMR spectra of the SM and SM-NP fractions (Fig 2, S3 Fig).

To demonstrate that the antibacterial activity observed in the SM-NP fraction of the ES-GBBr formulation originated from allicin, depletion experiments were conducted by addition of exogenous free cysteine to the SM-NP fraction. Allicin depletion within this fraction and an allicin standard was confirmed using 1D ^1^H NMR spectroscopy (S2 Fig) prior to evaluation of antibacterial activity against *S*. *aureus* strain LAC and *P*. *aeruginosa* strain PA215. The allicin-depleted SM-NP fraction demonstrated no observable antibacterial activity against the aforementioned bacterial strains with an MIC value greater than 40% (v/v); furthermore, this reflected an MIC increase greater than 16-fold for *S*. *aureus* strain LAC and greater than 4-fold for *P*. *aeruginosa* strain PA215 relative to control SM-NP fraction (Table 4).

**Table 4:** Effect of allicin depletion on susceptibility of multi-drug resistant bacterial strains to ES-GBBr formulation SM-NP fraction.

Bacterial strains	MIC^a^ (FC^b^)	
	Control	Allicin-Depleted
*S*. *aureus*		
LAC	2.5 (1)	>40 (>16)
*P*. *aeruginosa*		
PA215	10 (1)	>40 (>4)

MIC was determined using a broth microdilution method as described by the Clinical and Laboratory Standards Institute. Allicin depletion was conducted by addition of free cysteine and incubation for 1 hr at 4 °C prior to MIC evaluation. Control was not subjected to allicin depletion. Reported values represent the median of at least 3 biological replicates.

^a^MIC values indicate the concentration of ES-GBBr SM-NP fraction in % (v/v)

^b^FC values indicate the fold change in the MIC relative to that of ES-GBBr SM-NP fraction

### Allicin concentration and production within distinct Bald’s eyesalve formulations

**Fig 3:**
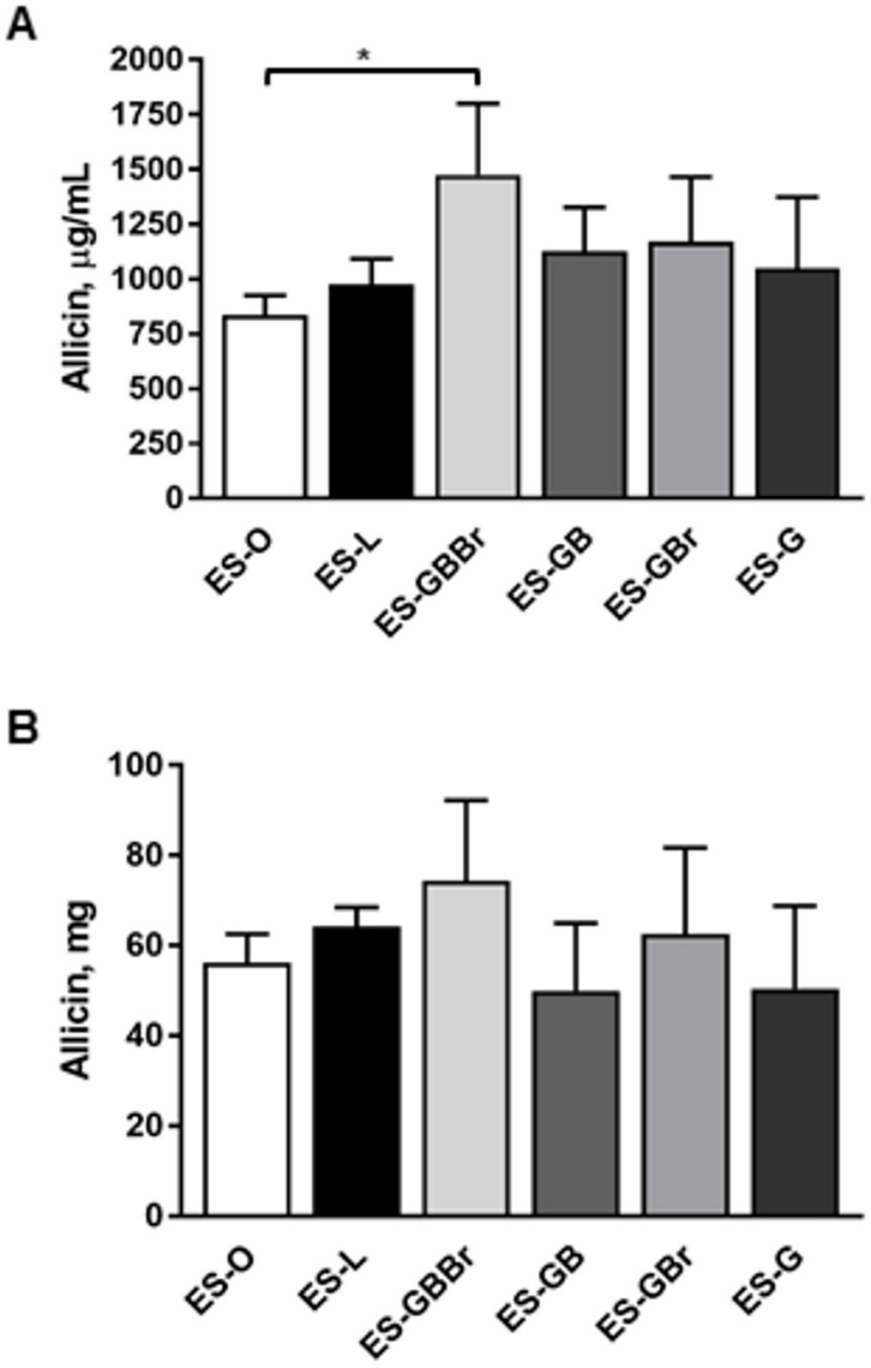
Quantitation of allicin in eyesalve formulations reveals significant differences in concentration but not overall yield. (A) Allicin concentration (μg/mL) was determined using Chenomx small-molecule library for 600 MHz (^1^H Larmor frequency) magnetic field strength NMR. (B) Allicin production (mg) was calculated by accounting for volume differences between formulations. Data are presented as mean ± SD (n = 4). Significant differences (*p* < 0.05, one-way ANOVA, Tukey’s multiple comparisons post-test) between formulations are indicated with asterisks.

To assess whether the enhanced antibacterial activity of the ES-GBBr formulation was due to a greater concentration of allicin, 1D ^1^H NMR and Chenomx NMR Suite software was used to quantify the concentration of allicin in all Bald’s eyesalve formulations described in Table 1. The ES-GBBr formulation exhibited a significantly greater concentration of allicin than the ES-O formulation, with mean values of 1476.6 and 836.2 μg/mL, respectively (Fig 3A). Although not significantly distinct from the ES-GBBr formulation, the average allicin concentration in the ES-L, ES-GB, ES-GBr, and ES-G formulations were 977.3, 1127.3, 1170.4, and 1050.6 μg/mL respectively (Fig 3A).

Due to variations in final volume resulting from ingredient omission(s), allicin yield (mg) was also calculated for each formulation. The average amount of allicin produced in the ES-O, ES-L, ES-GBBr, ES-GB, ES-GBr, and ES-G formulations was 56.2, 64.2, 74.4, 50.0, 62.5, and 50.4 mg respectively; however, there was no significant difference in the amount of allicin present between any of these formulations (Fig 3B).

### Cumulative effects regarding the antibacterial activity of allicin within Bald’s eyesalve against *S*. *aureus* and *P*. *aeruginosa*

To evaluate whether interactions between ingredients within the Bald’s eyesalve ES-GBBr formulation impact the efficacy of allicin against drug resistant phenotypes, the MIC values of an allicin standard and allicin within crude ES-GBBr were determined against *S*. *aureus* strain LAC and *P*. *aeruginosa* strain PA215. The allicin standard exhibited an MIC value of 12.2 μg/ml against *S*. *aureus* strain LAC and 97.4 μg/ml against *P*. *aeruginosa* strain PA215, while allicin within crude ES-GBBr displayed an MIC value of 24.3 μg/ml against *S*. *aureus* strain LAC and 48.7 μg/ml against *P*. *aeruginosa* strain PA215 (Table 5). These results suggested that additional components within the Bald’s eyesalve ES-GBBr formulation act in an antagonistic manner with the antibacterial activity of allicin against *S*. *aureus* strain LAC, but additively or synergistically against *P*. *aeruginosa* strain PA215 (Table 5).

**Table 5:** Evaluation of conjugative effects within crude ES-GBBr formulation against multi-drug resistant bacterial strains.

Bacterial strains	MIC^a^ (FC^b^)		Interaction
	Control	ES-GBBr	ES-GBBr
*S*. *aureus*			
LAC	12.2 (1)	24.3 (2)	Antagonistic
*P*. *aeruginosa*			
PA215	97.4 (1)	48.7 (-2)	Additive or Synergistic

MIC was determined using a broth microdilution method as described by the Clinical and Laboratory Standards Institute. Control MIC values were determined using an allicin standard. Reported values represent the median of at least 3 biological replicates.

^a^MIC values indicate the concentration of allicin in μg/mL

^b^FC values indicate the fold change in the MIC relative to control

## Discussion

In this study, we have shown that Bald’s eyesalve, an Anglo-Saxon remedy for eye sty infections, displays growth inhibitory activity against *S*. *aureus* and *P*. *aeruginosa*, including a multi-drug resistant strain. This activity was more pronounced against *S*. *aureus* than *P*. *aeruginosa*, which is consistent with the fact that Gram-negative bacteria, particularly *Pseudomonads*, are notably more resistant to antibacterial agents than Gram-positive bacteria, including *Staphylococcus sp*. [12]. Our data indicate that the specific formulation composition had little impact on the inhibition of *S*. *aureus*. This is inconsistent with a previous report where the presence of an additional *Allium* species, onion or leek, was found to significantly contribute to the antibacterial activity of Bald’s eyesalve [6]; however, this previous study evaluated the antibacterial activity using a synthetic wound model in which *S*. *aureus* inoculum was grown as a biofilm for 24 hr prior to treatment and based upon colony-forming units [6]. Our conflicting results may be due to our assessment of the antibacterial activity of Bald’s eyesalve exclusively against planktonic *S*. *aureus* rather than *S*. *aureus* biofilms, and we did not investigate whether onion or leek are necessary for specific efficacy against bacterial biofilms. Previous studies have shown that quercetin, an antibacterial flavonoid found in onion [13], and its derivatives demonstrate anti-biofilm and anti-quorum sensing activity against *S*. *aureus* and *P*. *aeruginosa* [14, 15]. Although plant extracts have been shown to display growth inhibitory activity against *P*. *aeruginosa* [16, 17], to our knowledge our study represents the first report of the antibacterial efficacy of Bald’s eyesalve against *P*. *aeruginosa*.

Although all known *Allium* species contain organosulfur compounds, the chemical composition and resultant antibacterial activity of their extracts greatly varies [18]. Prior investigations have determined the main constituents of garlic essential oil to be diallyl disulfide (DADS), diallyl trisulfide (DATS), allyl methyl trisulfide, diallyl sulfide (DAS), and diallyl tetrasulfide (DATTS), while the main constituents of onion and leek essential oils were found to be dipropyl disulfide, dipropyl trisulfide, methyl propyl disulfide, methyl propyl trisulfide, and 1-propenyl propyl disulfide [18, 19]. Tsao *et al*. previously demonstrated that DAS, DADS, DATS, and DATTS exhibit MICs of 20, 4, 2, and 0.5 μg/ml against *S*. *aureus*, respectively, and 80, 64, 32, and 12 μg/ml against *P*. *aeruginosa*, respectively [20, 21]. Little is known regarding the antibacterial activity of dipropyl disulfide and dipropyl trisulfide against *P*. *aeruginosa*; however, Kim *et al*. established the MICs of dipropyl disulfide and dipropyl trisulfide against *S*. *aureus* to be >500 μg/ml and 50 μg/ml, respectively [22]. These findings suggest that the antibacterial activity of the *Allium* species used in our study would be in the following order: garlic > onion > leek. However, our data indicate that addition of leek to Bald’s eyesalve results in greater antibacterial activity, in particular against clinical isolate strains, relative to the addition of onion (Table 2). Additional studies are necessary to elucidate the activity of organosulfur compounds derived from onion and leek, especially dipropyl disulfide and dipropyl trisulfide with regard to clinical isolates.

Crude botanical extracts that display antibacterial activity are often subjected to repetitive cycles of fractionation until relatively pure biologically active compounds are identified [23]. Many fractionation scheme variations have been utilized for the isolation of natural compounds, with each offering its own specific advantages [24, 25]. Sub-fractions generated are often less complex than the crude parent extract, thus simplifying the process of identifying the active component(s) [26]. We fractionated Bald’s eyesalve formulation ES-GBBr using a combination of molecular size exclusion and organic solvent partitioning methods to isolate and identify its principal antibacterial agent. A review of the literature suggests that our study is the first to characterize the activity of Bald’s eyesalve using fractionation in conjunction with growth inhibitory assays; however, there are several limitations to bioassay-guided fractionation. These include an inherent bias towards the most dominant bioactive compounds in the extract or fraction, which can lead to a disregard of low abundant, yet biologically active, components [27]. In addition, activity may be lost during fractionation, particularly if the bioactive agent is temperature-sensitive or binds to the chromatographic resins employed [28, 29]. Due to the instability of allicin at room temperature and in organic solvents [30, 31], a significant amount of allicin may have been lost from our ES-GBBr formulation SM-NP fraction as suggested by the NMR spectral features shown in Fig 1. In an attempt to reduce activity loss, we stored our ES-GBBr formulation SM-NP fraction in water. This could explain the 2-fold MIC increase observed for all bacterial strains upon exposure to the SM-NP fraction relative to the SM fraction of our ES-GBBr formulation (Table 3); furthermore, these observations reinforce our findings that allicin is the dominant antibacterial agent in Bald’s eyesalve.

This study identified allicin as the principal antibacterial compound present in Bald’s eyesalve. Allicin (3-[(prop-2-ene-1-sulfinyl)sulfanyl]prop-1-ene) is a reactive sulfur species that is used as a defense mechanism in garlic following tissue damage [32] and is known to be the most significant bioactive component within fresh garlic extract; furthermore, allicin has been shown to exhibit antibacterial activity against a diverse range of both Gram-positive and Gram-negative bacteria, including *Streptococcus* spp., methicillin-sensitive and methicillin-resistant *S*. *aureus*, *Salmonella typhimurium*, *Escherichia coli*, and *Vibrio cholerae* [33–36]. Previous studies have demonstrated that the mechanism of action of allicin is highly dependent upon the capacity of its thiosulfinate group to alter the intracellular glutathione pool and inhibit metabolic and oxidative stress enzymes by oxidation of thiols within cells [34, 37]. Unlike most modern clinical antibiotics, allicin has an inherent advantage in that it does not target any specific cellular component; therefore, the development of resistance by targeted mutation is improbable, making allicin particularly suitable for application against drug resistant bacteria [34]. We exploited the thiol-reactive nature of allicin to facilitate its depletion from the ES-GBBr formulation SM-NP fraction by addition of exogenous free cysteine and found that there was no observable antibacterial activity against either *S*. *aureus* or *P*. *aeruginosa* following depletion of allicin. These results identify allicin as the primary antibacterial compound present in Bald’s eyesalve.

Considering that the primary targets of allicin are intracellular thiols, such as those found in reduced glutathione, we evaluated the concentration and yield of allicin between several Bald’s eyesalve formulations. Our results indicated that there was a significant difference in the concentration of allicin present in the ES-GBBr formulation when compared to the ES-O formulation (Fig 3A); therefore, the enhanced antibacterial activity demonstrated by our ES-GBBr formulation can be attributed to a greater concentration of allicin. Consequently, we assessed total mg of allicin in all of our Bald’s eyesalve formulations to determine whether distinct ingredient combinations were more favorable for allicin production. Based upon previous studies demonstrating aqueous ethanol to be a superior extraction solvent for allicin [30, 38], we conclude that allicin is extracted from minced garlic in our Bald’s eyesalve formulations and stabilized by the presence of ethanol from the addition of wine, which was determined to be 3–4% v/v (S1 Table). Although there was no statistically significant difference in the yield of allicin among our formulations (Fig 3B), the formulations containing brass (ES-O, ES-L, ES-GBBr, and ES-GBr) amassed on average more allicin than those that omitted this ingredient (ES-GB and ES-G). In particular, the ES-GBBr formulation accrued the most allicin at 74.4 mg. These results suggest that the combination of brass and bovine bile in Bald’s eyesalve may be favorable for extraction of allicin from minced garlic. A previous study demonstrated that alliinase, the enzyme responsible for the conversion of alliin to allicin, displays increased activity in the presence of metal ions, most notably Fe^2+^, Mn^2+^, and Zn^2+^ [39]. We hypothesize that dezincification [40, 41] of the brass 260 alloy included in several of our formulations takes place due to the mildly acidic pH of Bald’s eyesalve (S1 Table) which may cause corrosion. In addition, we suspect that bile salts act as a metal ion buffer in Bald’s eyesalve, increasing dezincification and solubility of Zn^2+^ by the formation of micellar complexes [42]. Future work is needed to identify the chemical processes by which brass and bile interact to alter allicin production in Bald’s eyesalve.

While ethanol is a well-known and widely used disinfectant, its growth inhibitory activity due to the addition of wine in Bald’s eyesalve can be considered minimal. Prior studies have established the MIC and minimum bactericidal concentration (MBC) for ethanol against *S*. *aureus* to be 6.25% and 25% (v/v), respectively, and against *P*. *aeruginosa* to be 1.56% and >12.5% (v/v), respectively [43, 44]. Given the concentration of ethanol in our Bald’s eyesalve formulations (S1 Table) and their corresponding MIC values (Table 2), we determined that ethanol varied from 0.02 to 0.40% (v/v) at growth inhibitory concentrations of Bald’s eyesalve. At bactericidal concentrations, ethanol can perturb the structure, function, pH gradient (ΔpH), membrane potential (ΔΨ), and composition of cellular membranes [45, 46], inhibit cell division and nutrient transport [45, 47], and reduce intracellular pH [47, 48]. Since ethanol is present in such low concentrations within our Bald’s eyesalve formulations, we conclude that any contribution ethanol could make to the overall antibacterial activity, aside from promoting the extraction and stabilization of allicin, is negligible.

In an attempt to mitigate the increasing prevalence of drug resistant bacterial infections, treatment regimens including multiple antibiotics are widely utilized. Antibiotic combinations can generate additive or synergistic effects that enhance antibacterial activity and/or reduce the dosage required to eradicate infection [49, 50]; in addition, using multiple antibiotics with distinct mechanisms of action reduces the risk of progressive resistance development following treatment [51]. A recent study suggested that the antibacterial efficacy of Bald’s eyesalve may be due to the combined activity of compounds arising from several ingredients in the formulation, since it was determined that garlic, wine, and an additional *Allium* species were essential to obtain full activity [6]. Our study revealed that the principal antibacterial agent within Bald’s eyesalve is allicin derived from garlic; however, our MIC data also indicate that the allicin present in this medieval remedy exhibits antagonistic antibacterial activity with other compounds present in the formulation when used against *S*. *aureus*, whereas it displays additive or synergistic activity against *P*. *aeruginosa*. Our data thus support the notion that the combinatorial formulation of Bald’s eyesalve is not a more effective means of treatment than allicin alone against *S*. *aureus*, whereas it is more effective against *P*. *aeruginosa*. Previous studies have shown that combining allicin with additional antifungal and antibacterial agents, such as amphotericin B and silver nanoparticles, results in synergistic activity [52, 53]; however, we have yet to identify the accessory agent(s) present in Bald’s eyesalve that results in additional activity with allicin when used against *P*. *aeruginosa*. This study highlights the importance of evaluating cumulative effects of antibiotics within complex mixtures and multi-drug treatment regimens, particularly when the target bacteria responsible for the infection have been identified [54, 55].

## Conclusions

Results from this study demonstrated that Bald’s eyesalve possesses antibacterial activity against both Gram-positive and Gram-negative bacteria including phylogenetically distinct strains of *S*. *aureus* and *P*. *aeruginosa* that exhibit variable degrees of antibiotic resistance and virulence. In addition, our study identified allicin as the principal antibacterial agent present in Bald’s eyesalve and determined that its activity among additional formulation components is antagonistic against *S*. *aureus*, whereas it acts in an additive or synergistic fashion with other Bald’s eyesalve ingredients when used against *P*. *aeruginosa*. These results emphasize the value of establishing the source of antibacterial activity present in plant-derived natural products for therapeutic use, including how to use complex natural mixtures to treat drug resistant bacterial infections. Furthermore, our findings underline the importance of assessing the additive, synergistic, and/or antagonistic effects of combining different ingredients within these ancient formulae as these have significant consequences with regards to the antibacterial efficacy of these natural remedies.

## Supporting information

S1 Fig ^1^H NMR analysis of ES-GBBr formulation fractions. Stacked 1D ^1^H NMR spectra (600 MHz, 10% D_2_O) in chemical shift regions (A) 0–5 ppm and (B) 5–10 ppm for ES-GBBr SM (black), SM-P (red), and SM-NP (blue) fractions. Insets display the expanded 1D ^1^H NMR spectrum in chemical shift regions (A) 0.5–4.5 ppm and (B) 5.1–8.5 ppm for the ES-GBBr SM-NP (blue) fraction.
(TIF)

S2 Fig Thiol-reactive nature of allicin is exploited to facilitate depletion from ES-GBBr formulation SM-NP fraction. An overlay of 1D ^1^H NMR spectra (600 MHz, 10% D_2_O) in chemical shift region 5.1–6.1 ppm for (A) an allicin standard and (B) ES-GBBr formulation SM-NP fraction with (green/red) and without (black/blue) addition of exogenous free cysteine, respectively, followed by 4 °C incubation for 1 hr.
(TIF)

S3 Fig Allicin structure and ^1^H NMR spectral data. The molecular structure of allicin is shown in (A). Assignments, chemical shifts (δ, ppm), relative integrated intensities, multiplicities, and coupling constants (J, Hz) from ^1^H NMR spectra are displayed for (B) an allicin standard and (C) allicin in ES-GBBr formulation SM-NP fraction.
(TIF)

S1 Table pH and ethanol % v/v of Bald’s eyesalve formulations. (XLSX)
