# Supplementary figures and images for: Characterization of the antibacterial activity of Bald’s eyesalve against drug resistant Staphylococcus aureus and Pseudomonas aeruginosa
Source: PLoS One. 2018 Nov 28;13(11):e0208108. doi: 10.1371/journal.pone.0208108 (PMC6261618; doi:10.1371/journal.pone.0208108)

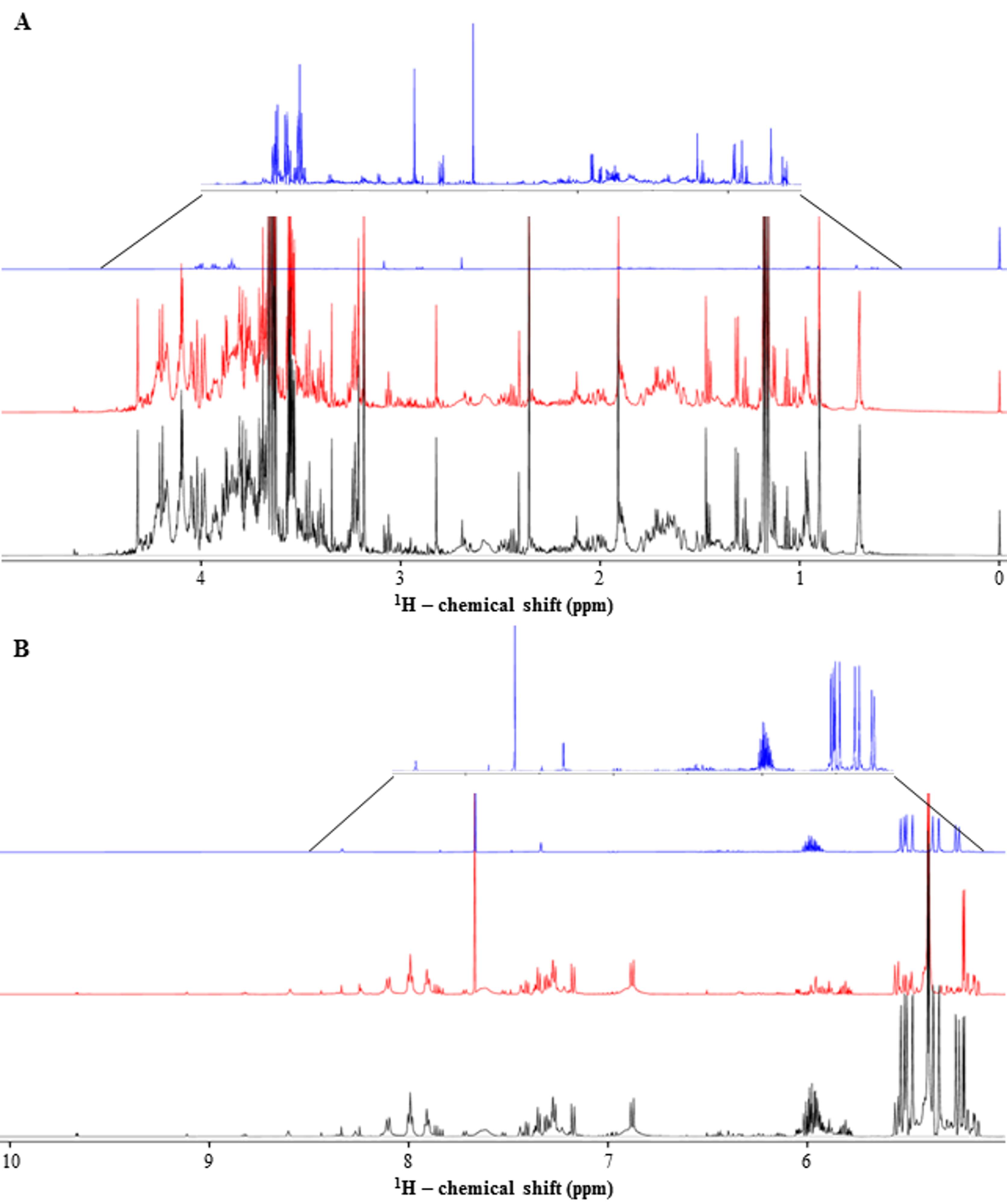

Supplement: S1 Fig — Stacked 1D 1H NMR spectra (600 MHz, 10% D2O) in chemical shift regions (A) 0–5 ppm and (B) 5–10 ppm for ES-GBBr SM (black), SM-P (red), and SM-NP (blue) fractions. Insets display the expanded 1D 1H NMR spectrum in chemical shift regions (A) 0.5–4.5 ppm and (B) 5.1–8.5 ppm for the ES-GBBr SM-NP (blue) fraction. (TIF) [file pone.0208108.s001.tif]

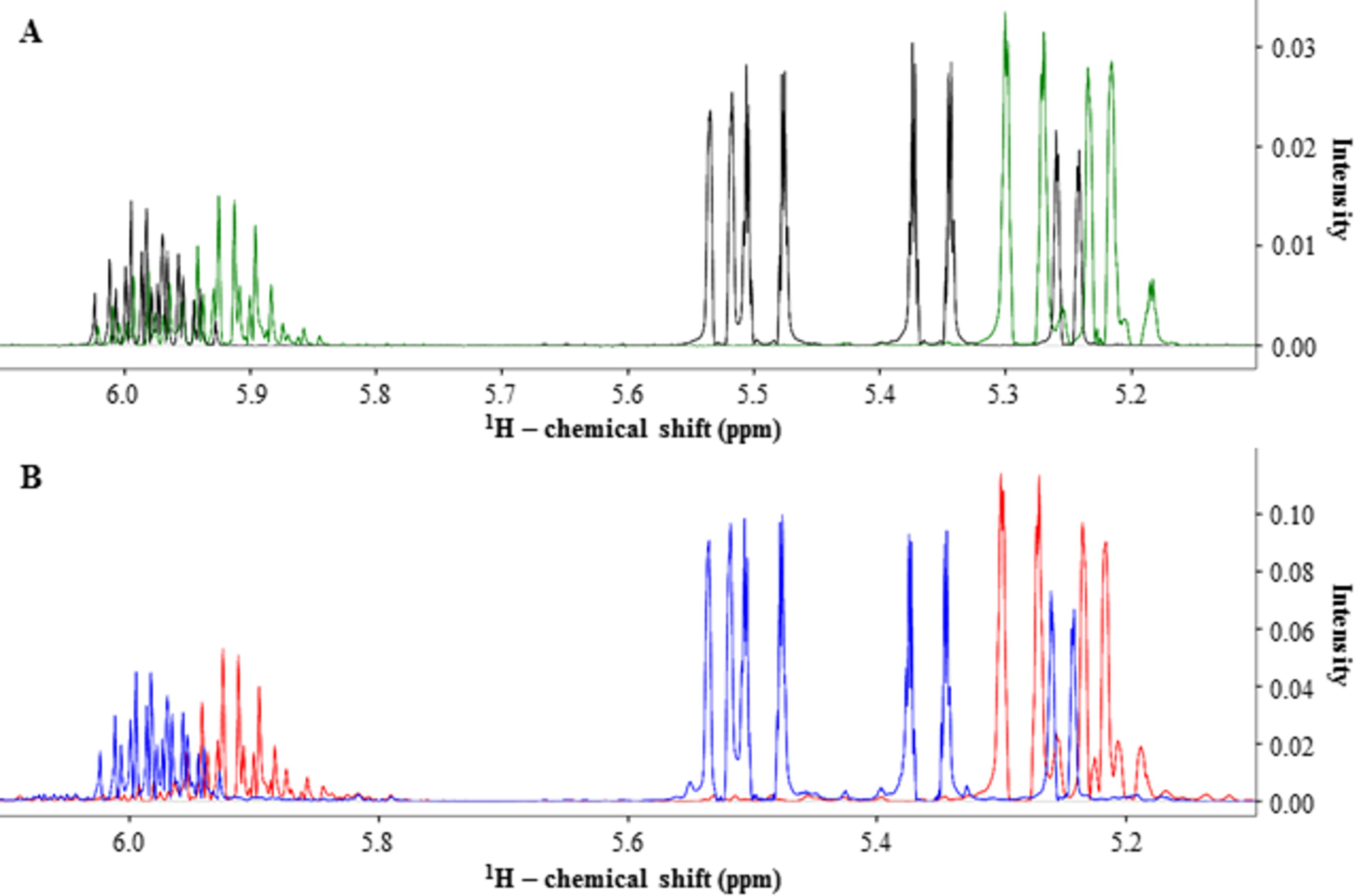

Supplement: S2 Fig — An overlay of 1D 1H NMR spectra (600 MHz, 10% D2O) in chemical shift region 5.1–6.1 ppm for (A) an allicin standard and (B) ES-GBBr formulation SM-NP fraction with (green/red) and without (black/blue) addition of exogenous free cysteine, respectively, followed by 4 °C incubation for 1 hr. (TIF) [file pone.0208108.s002.tif]

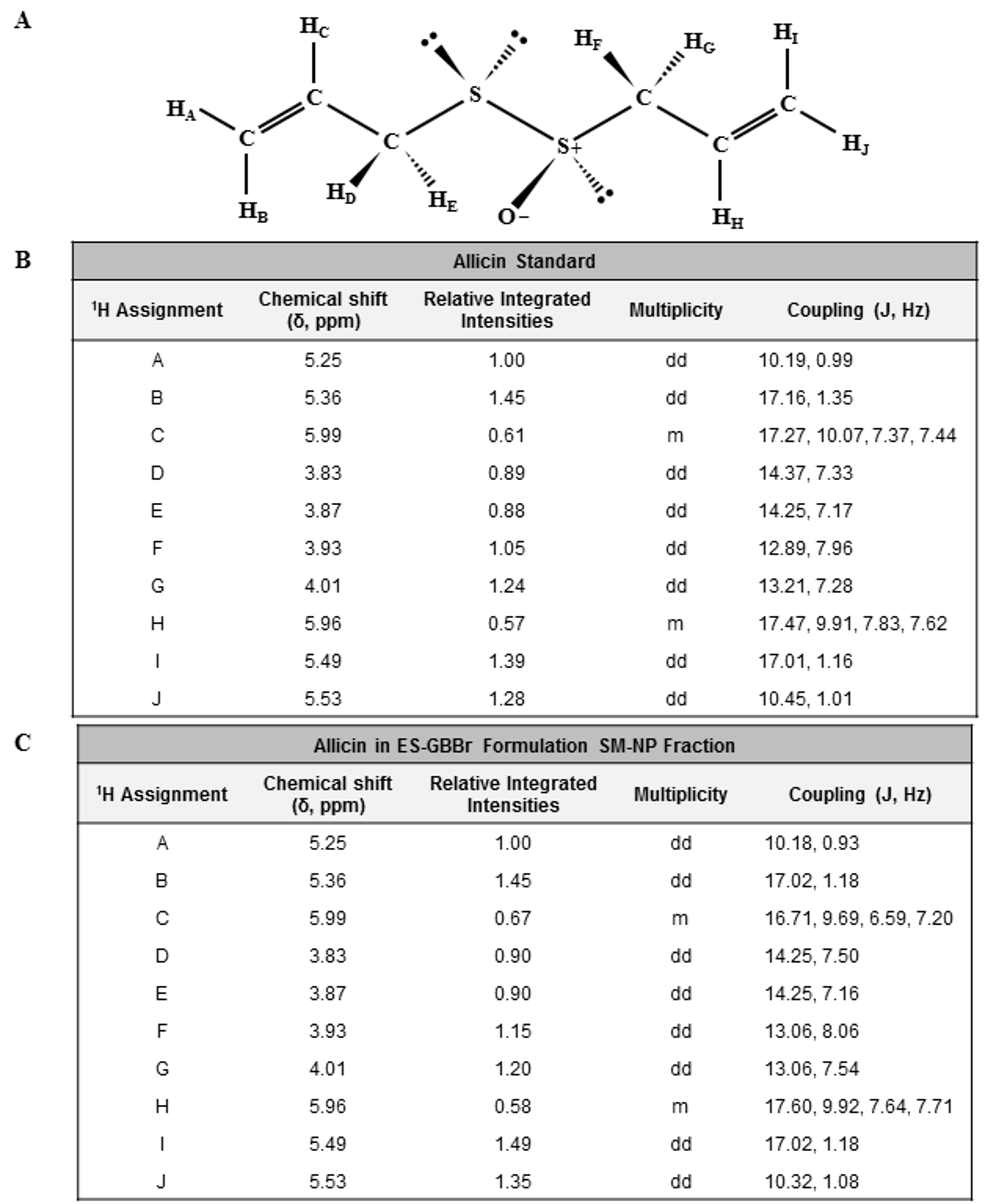

Supplement: S3 Fig — The molecular structure of allicin is shown in (A). Assignments, chemical shifts (δ, ppm), relative integrated intensities, multiplicities, and coupling constants (J, Hz) from 1H NMR spectra are displayed for (B) an allicin standard and (C) allicin in ES-GBBr formulation SM-NP fraction. (TIF) [file pone.0208108.s003.tif]
